# Blood flow speed of the gastric conduit assessed by indocyanine green fluorescence: New predictive evaluation of anastomotic leakage after esophagectomy

**DOI:** 10.1097/MD.0000000000004386

**Published:** 2016-07-29

**Authors:** Kazuo Koyanagi, Soji Ozawa, Junya Oguma, Akihito Kazuno, Yasushi Yamazaki, Yamato Ninomiya, Hiroki Ochiai, Yuji Tachimori

**Affiliations:** a Division of Esophageal Surgery; b Division of Colorectal Surgery, Department of Gastrointestinal Oncology, National Cancer Center Hospital, Tokyo; c Department of Gastroenterological Surgery, Tokai University School of Medicine, Isehara, Japan.

**Keywords:** anastomotic leakage, blood flow speed, esophagectomy, gastric conduit, indocyanine green fluorescence

## Abstract

Anastomotic leakage is considered as an independent risk factor for postoperative mortality after esophagectomy, and an insufficient blood flow in the reconstructed conduit may be a risk factor of anastomotic leakage. We investigated the clinical significance of blood flow visualization by indocyanine green (ICG) fluorescence in the gastric conduit as a means of predicting the leakage of esophagogastric anastomosis after esophagectomy.

Forty patients who underwent an esophagectomy with gastric conduit reconstruction were prospectively investigated. ICG fluorescence imaging of the gastric conduit was detected by a near-infrared camera system during esophagectomy and correlated with clinical parameters or surgical outcomes.

In 25 patients, the flow speed of ICG fluorescence in the gastric conduit wall was simultaneous with that of the greater curvature vessels (simultaneous group), whereas in 15 patients this was slower than that of the greater curvature vessels (delayed group). The reduced speed of ICG fluorescence stream in the gastric conduit wall was associated with intraoperative blood loss (*P* = 0.008). Although anastomotic leakage was not found in the simultaneous group, it occurred in 7 patients of the delayed group (*P* < 0.001). A flow speed of ICG fluorescence in the gastric conduit wall of 1.76 cm/s or less was determined by a receiver operating characteristic (ROC) curve, identified as a significant independent predictor of anastomotic leakage after esophagectomy (*P* = 0.004).

This preliminary study demonstrates that intraoperative evaluation of blood flow speed by ICG fluorescence in the gastric conduit wall is a useful means to predict the risk of anastomotic leakage after esophagectomy.

## 1 Introduction

Esophagectomy with 3-field lymph node dissection is performed as a standard surgical procedure for localized thoracic esophageal cancer in Japan.^[1,2]^ Morbidity is a major concern during the follow-up period because of the invasive nature of this surgery and the complex operative procedures involved.^[3,4]^ The anastomosis between the cervical esophagus and gastric conduit, commonly used for the reconstruction after esophagectomy, is more likely to leak than other gastrointestinal anastomosis and is consequently associated with higher postoperative mortality.^[5,6]^ Among several factors, such as preoperative nutritional status, reconstructed route, and site or technique of the anastomosis,^[7–9]^ there is a high probability that ischemia of the gastric conduit can attribute to the anastomotic leakage.^[10]^ Perfusion and viability of the conduit has been subjectively determined by clinical judgment, such as the color, movement, and pulsation of the vessels. If substantial intraoperative measurement system of tissue blood flow is established, the surgeons can obtain objective and reliable information to decide the appropriate anastomotic site after esophagectomy. Laser Doppler flowmetry, which can detect microperfusion at any point on the gastric conduit surface, emerged as a promising tissue blood flow assessment system. However, it has not been widely accepted by surgeons due to the lack of reproducibility and no apparent correlation with anastomotic leakage.^[11,12]^ A new assessment system that can indicate not only microperfusion, but also macroperfusion of the gastric conduit and that can predict the risk of the leakage of esophagogastric anastomosis is urgently recommended.

Near-infrared fluorescence using indocyanine green (ICG) has been applied as a real-time navigation tool in various surgical fields.^[13–16]^ ICG is a water-soluble near-infrared fluorophore, with its immediate or long-term safety confirmed by previous studies.^[17,18]^ ICG fluorescence imaging provides high sensitivity and significant contrast because of its low inherent autofluorescence background and high tissue penetration.^[19]^ Recent studies have shown that ICG fluorescence can visualize the blood flow of the gastric conduit in patients undergoing esophagectomy; however, the correlation between the blood flow observed by ICG fluorescence and the occurrence of esophagogastric anastomotic leakage has not been demonstrated.^[20–22]^ To evaluate the usefulness of ICG fluorescence in the gastric conduit, there are several issues to be considered, including the observation site, detection time, and the parameters and methods of ICG fluorescence imaging. Furthermore, to our knowledge, the factors that may change tissue blood flow in the gastric wall (e.g., prior therapy, mucosal atrophy, gastric ulcer, and smoking) or that may change ICG fluorescence intensity (e.g., ICG concentration, binding properties, and interfering substances) have not been correlated with ICG fluorescence imaging of the gastric conduit in previous studies.

In this study, we aimed to establish novel methods to evaluate the blood flow using ICG fluorescence in the wall of the gastric conduit during esophagectomy. We first investigated associations between the ICG fluorescence stream and various patient clinical factors. We then investigated the significance of the flow speed of ICG fluorescence in the gastric conduit as a means of predicting esophagogastric anastomotic leakage after esophagectomy.

## 2 Methods

### 2.1 Patients

This study was investigated to develop new methods that could assess the blood flow of the gastric conduit using ICG fluorescence. The duration of the study was planned for 2 years. So the number of patients we could enroll in the study was calculated as 50 from annual surgical cases of the esophagectomy with 3-field lymph node dissection in Tokai University Hospital. Consequently, we prospectively and consecutively enrolled 46 esophageal cancer patients in the study between January 2014 and October 2015. Among them, ICG fluorescence was performed in 40 patients; the remaining 6 were excluded because of iodine hypersensitivity (4 patients), reconstruction using the small intestine (1 patient), and exploratory thoracotomy (1 patient). The characteristics of the patients included in the study are summarized in Table 1. The cancer stage was determined according to the seventh edition of the International Union Against Cancer TNM Classification of Malignant Tumors.^[23]^ The study was approved and carried out according to guidelines set forth by the Institutional Review Board for Clinical Research of the Tokai University School of Medicine. All patients signed informed consent forms for operative procedures, injection of ICG, and observation of ICG fluorescence during the operation.

**Table 1:**
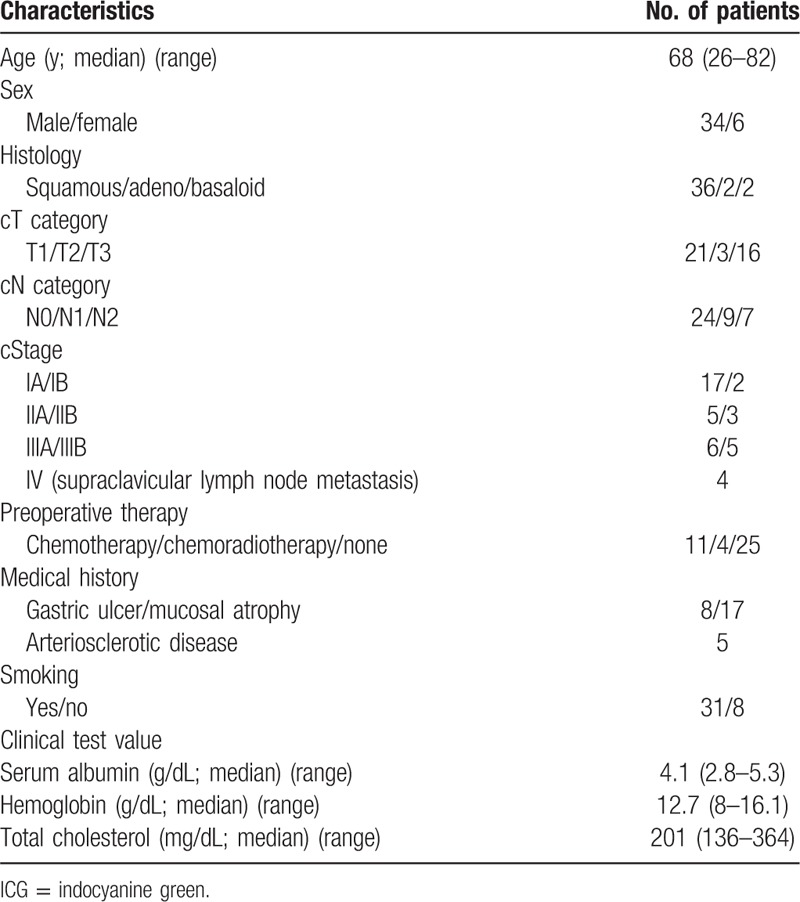
Patient characteristics in ICG fluorescence study.

### 2.2 Preparation of the gastric conduit and definition of anastomotic leakage

All patients received the same surgical procedures: resection of the esophagus with 3-field lymph node dissection, simultaneous reconstruction using a gastric conduit, and cervical anastomosis. After esophagus was isolated and resected, we prepared a gastric conduit with a width of 3.5 cm. The right gastric artery was preserved, and resection of lesser curvature was routinely determined at 5 cm from the pylorus. The right gastroepiploic artery and branches of the left gastroepiploic artery were also preserved. The gastric conduit was pulled up to the neck via the retrosternal, subcutaneous, and posterior mediastinal routes in 30, 7, and 3 patients, respectively. We routinely used the retrosternal route. Subcutaneous route was selected when the patient had a surgical risk such as salvage esophagectomy, and posterior mediastinal route was selected when the retrosternal route could not be used for some reasons such as prior operation or intervention to the cardiac diseases. Anastomosis between esophagus and the gastric conduit was performed in the cervical region. A circular stapler technique was used for the cases in 2014 (n = 12), and a hand-sewing method was used for those in 2015 (n = 28). We changed the anastomotic technique from circular stapler to hand-sewing because we experienced some difficulties of the technique and an adverse event caused by mechanical trouble of circular stapler in other cases. No patients underwent additional arterial or venous microvascular anastomosis between gastric conduit and cervical vessels.

Postoperative esophagography was performed to check the integrity of the anastomosis in all patients. Besides radiological diagnosis, clinical findings such as saliva discharge from the incised wound were defined as anastomotic leakage.

### 2.3 ICG fluorescence imaging

The gastric conduit was stretched, pulled up to the neck, and placed on the anterior chest wall, as assuming the anastomosis. After checking the gastric conduit for its conditions, such as the color of the gastric serosa, arterial pulsation of the gastroepiploic artery, and connection between the right and left gastroepiploic vessels, 2.5 mg (n = 24) or 1.25 mg (n = 16) of ICG dye (Diagnogreen; Daiichi-Sankyo Pharmaceutical, Tokyo, Japan) was injected as a bolus via a central venous catheter. ICG fluorescence was detected by a near-infrared camera system (Photodynamic Eye [PDE]; Hamamatsu Photonics K. K., Hamamatsu, Japan). ICG fluorescence imaging was performed using a charge-coupled device camera, comprising a light-emitting diode at a wavelength of 760 nm as a light source (activation of ICG) and a filter to eliminate light of wavelengths below 820 nm (detection of ICG fluorescence).^[13]^ The camera was held in the surgeon's hand and placed 20 cm above the gastric conduit. The fluorescence signals were sent to a digital video processor, displayed on a TV monitor, and were recorded.

**Figure 1:**
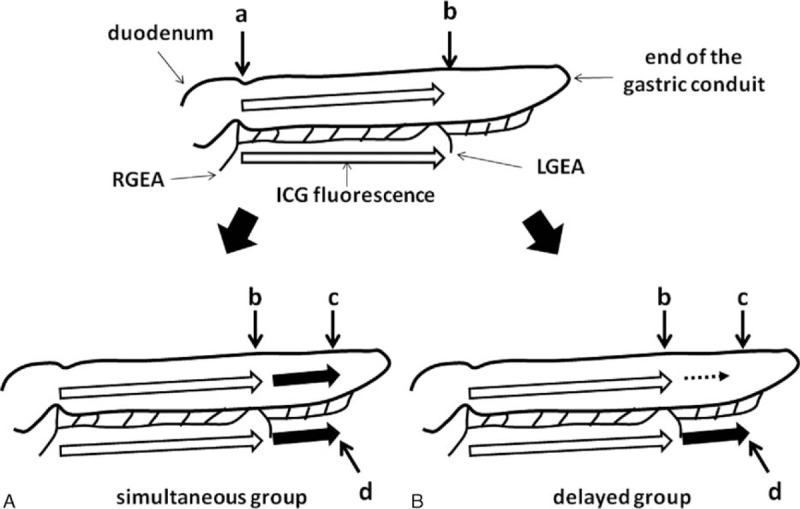
Schema of the indocyanine green (ICG) fluorescence stream of the gastric conduit. ICG fluorescence stream in the simultaneous group (A) and in the delayed group (B). Point **a**; ICG fluorescence at the root of right gastroepiploic artery and at the pylorus, point **b**; ICG fluorescence stream at terminal point of arterial pulsation of the greater curvature, point **c**; end of ICG fluorescence of the gastric conduit wall, point **d**; end of ICG fluorescence of the greater curvature vessels. LGEA = left gastroepiploic artery, RGEA = right gastroepiploic artery.

We selected 4 points to assess the ICG fluorescence of the gastric conduit. Point **a** was the pylorus, and point **b** was the terminal point of the gastroepiploic arterial pulsation. Terminal end of ICG fluorescence of the gastric conduit wall and greater curvature vessels were defined as point **c** and point **d**, respectively (Fig. 1). The flow speed of ICG fluorescence of the gastric conduit was calculated from the distance and moving time of ICG fluorescence between the points and was compared with clinical and surgical factors.

### 2.4 Statistical analysis

The parameters of ICG fluorescence imaging were compared with patient characteristics using a chi-square test and the Fisher exact test. The Mann–Whitney *U* test was used to assess the association between the continuous variables and ICG fluorescence imaging. A multinomial logistic regression analysis was used for predictive variable selection of the occurrence of anastomotic leakage after esophagectomy. A receiver operating characteristic (ROC) curve was generated for deciding the cutoff value of ICG fluorescence stream variables. Analysis was performed using JMP software (version 12; SAS Institute, Cary, NC). All tests were two-sided with a significance level <0.05.

## 3 Results

### 3.1 Intraoperative observation and ICG fluorescence imaging of the gastric conduit

The length of the gastric conduit (from the pylorus to the end of gastric conduit) was 34.9 ± 3.6 cm (mean ±  standard deviation [SD]), and this was not associated with the patient's height (*P* = 0.60) or weight (*P* = 0.36). The distance from point **a** to point **b** was 23.1 ± 3.1 cm, which was significantly associated with connection between the right and left gastroepiploic arteries: 25.0 ± 2.4 cm in patients with the connection and 22.2 ± 3.0 cm in those without the connection (*P* = 0.006).

Following ICG bolus injection, ICG fluorescence could be detected at the root of the right gastroepiploic artery within 30 seconds (point **a**). The arterial systolic blood pressure was 102.5 ± 12.3 mm Hg during ICG injection. ICG fluorescence in the wall of the gastric conduit subsequently moved with the blood flow, along with that of gastroepiploic artery, until it arrived at point **b**. ICG fluorescence of both the gastric conduit wall and the greater curvature vessels flowed 10.0 cm (point **c**) and 7.8 cm (point **d**) beyond point **b**, respectively (Table 2). The distance from point **a** to point **c** was also significantly associated with connection between right and left gastroepiploic arteries: 35.2 ± 3.6 cm in patients with the connection and 32.2 ± 3.4 cm in those without the connection (*P* = 0.014). The distance from point **a** to point **d** again showed a tendency to be associated with the connection between right and left gastroepiploic arteries: 33.0 ± 5.4 cm in patients with the connection and 29.8 ± 4.7 cm in those without the connection (*P* = 0.055).

**Table 2:**
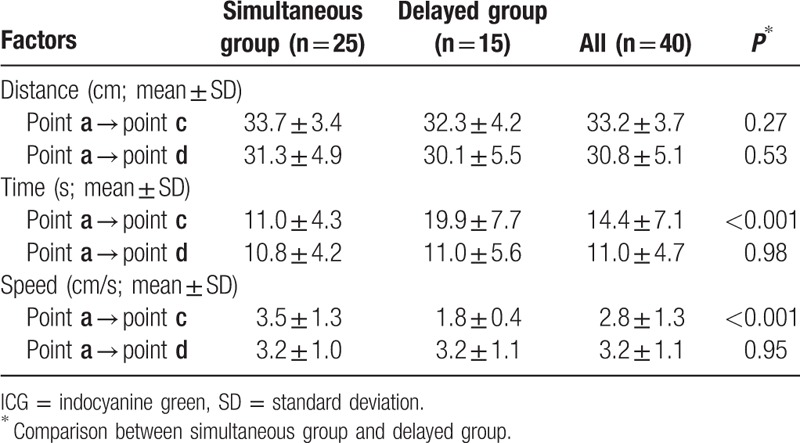
ICG fluorescence blood flow of the gastric conduit.

**Figure 2:**
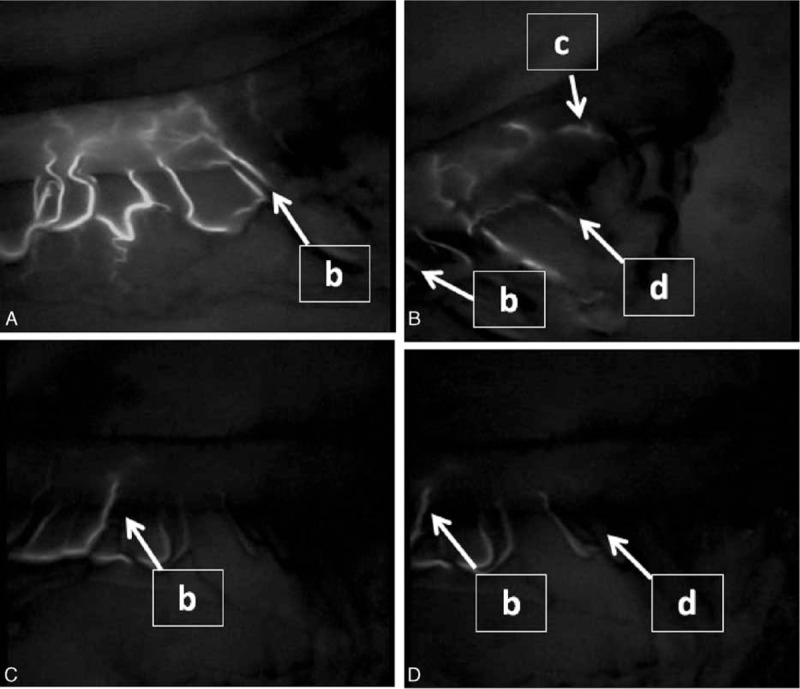
Representative indocyanine green fluorescence stream of the simultaneous group (A and B) and the delayed group (C and D).

Interestingly, we found that the flow speed of ICG fluorescence in the gastric conduit wall was not always similar to that of the greater curvature vessels beyond point **b** (Fig. 1). We focused on this ICG fluorescence stream in the gastric conduit wall and classified it into 2 groups according to the flow speed: a simultaneous group (n = 25), in which ICG fluorescence of the gastric conduit wall was almost the same as that of the greater curvature vessels beyond point **b** (Fig. 1, Fig. 2A and B), and a delayed group (n = 15), in which ICG fluorescence of the gastric conduit wall was obviously slower than that of the greater curvature vessels beyond point **b** (Fig. 1, Fig. 2C and D). The distance from point **a** to point **c** was not different between the simultaneous group and the delayed group. However, the ICG fluorescence stream from point **a** to point **c** took a significantly longer time in the delayed group than in the simultaneous group (*P* < 0.001). The flow speed of ICG fluorescence in the gastric conduit wall from point **a** to point **c** was 3.5 ± 1.3 cm/s in the simultaneous group and 1.8 ± 0.4 cm/s in the delayed group, respectively (*P* < 0.001). On the other hand, there were no differences in the distance and the flow speed of ICG fluorescence from point **a** to point **d** between the simultaneous group and the delayed group.

### 3.2 Comparison of the factors according to the ICG fluorescence speed in the gastric conduit wall

#### 3.2.1 Preoperative factors

Age, sex, medical histories, smoking, and preoperative therapies, which can each conceivably change the blood flow of the gastric conduit, were not associated with the flow speed of ICG fluorescence in the gastric conduit wall (Table 3). Similarly, blood chemistry results and ICG concentrations that might change the intensity of ICG fluorescence were not associated with the flow speed of ICG fluorescence.

**Table 3:**
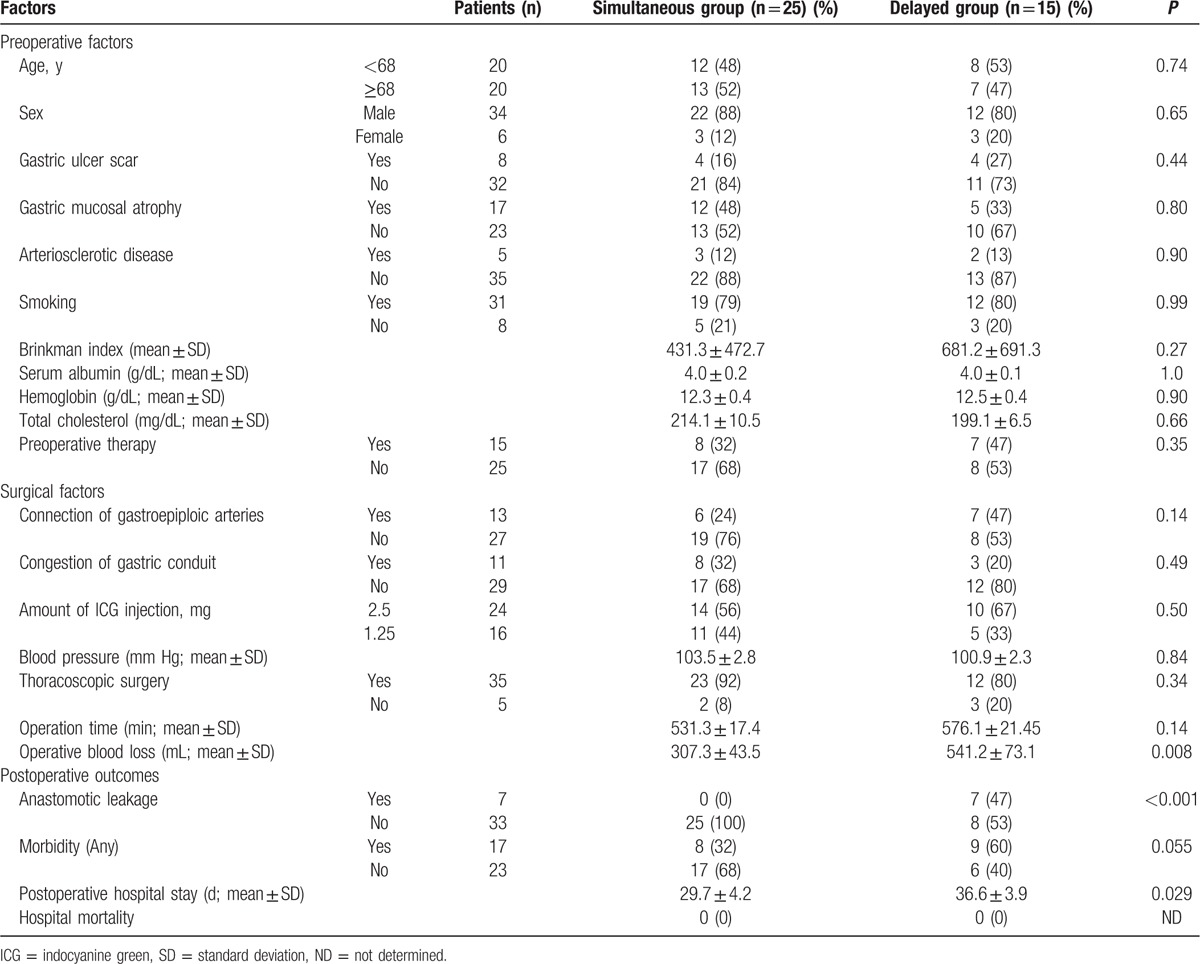
Comparison of clinical factors and surgical outcomes between the simultaneous group and the delayed group.

#### 3.2.2 Surgical factors

Although the distance from point **a** to point **c** was significantly different between patients with the connection of right and left gastroepiploic arteries and those without the connection, the flow speed of ICG fluorescence in the gastric conduit wall was not different (*P* = 0.14). Similarly, the congestion at the tip of the gastric conduit wall did not change the flow speed of ICG fluorescence in the gastric conduit wall (*P* = 0.41). Neither the amount of ICG injection nor the arterial blood pressure during ICG fluorescence imaging was associated with the flow speed of ICG fluorescence. Operation time was not significantly different between the simultaneous group and the delayed group (*P* = 0.14), whereas the intraoperative blood loss of the delayed group was significantly higher than that of the simultaneous group (*P* = 0.008).

#### 3.2.3 Postoperative outcomes

Although the anastomotic site was determined at the area in which ICG fluorescence was detected, anastomotic leakage was found in 7 patients. All of the anastomotic leakages occurred in the delayed group, with none in the simultaneous group (*P* < 0.001). Incidence of any morbidity (including anastomotic leakage) was higher in the delayed group (60%) than in the simultaneous group (32%), although this was not statistically significant (*P* = 0.055). The postoperative hospital stay of the delayed group was significantly longer than that of the simultaneous group (*P* = 0.029). Hospital mortality was not seen in either group.

### 3.3 Risk assessment for the anastomotic leakage

**Figure 3:**
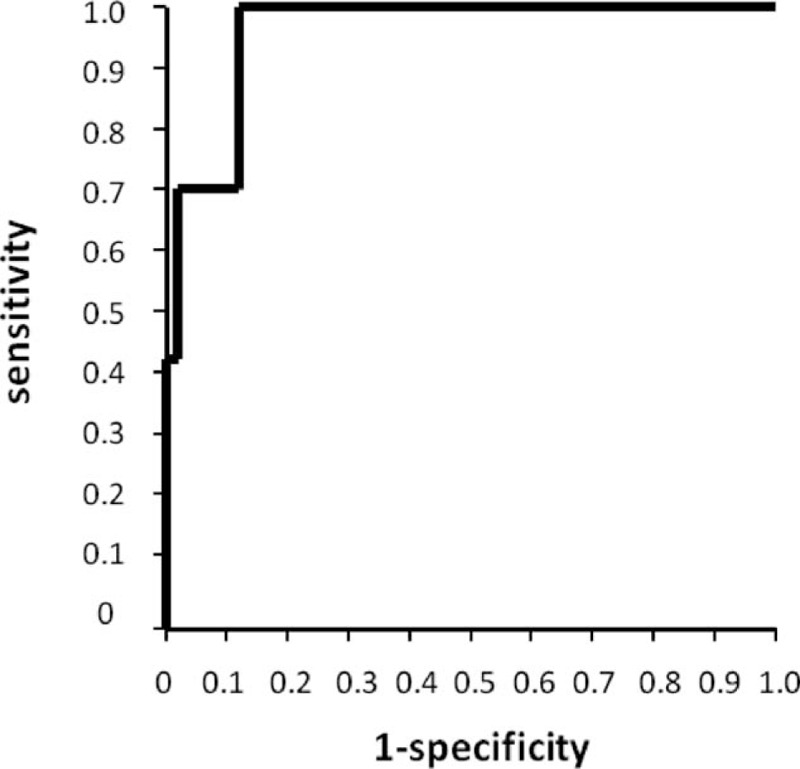
Receiver operating characteristic (ROC) curve of the speed of indocyanine green fluorescence stream in the gastric conduit wall was generated. Area under the curve was 0.96, and cutoff value was determined as 1.76 cm/s according to the ROC curve.

In subsequent analyses, the association between clinical parameters and occurrence of anastomotic leakage was assessed (Table 4). Both the preoperative and surgical factors were not correlated with the anastomotic leakage. Only the flow speed of ICG fluorescence in the gastric conduit wall from point **a** to point **c** was significantly correlated with the occurrence of anastomotic leakage after esophagectomy (*P* = 0.002). An ROC curve of the flow speed of ICG fluorescence in the gastric conduit wall was generated to predict the anastomotic leakage (Fig. 3). The cutoff value of the flow speed of ICG fluorescence on the gastric conduit wall was determined as 1.76 cm/s with an area under the curve of 0.96 according to the ROC curve.

**Table 4:**
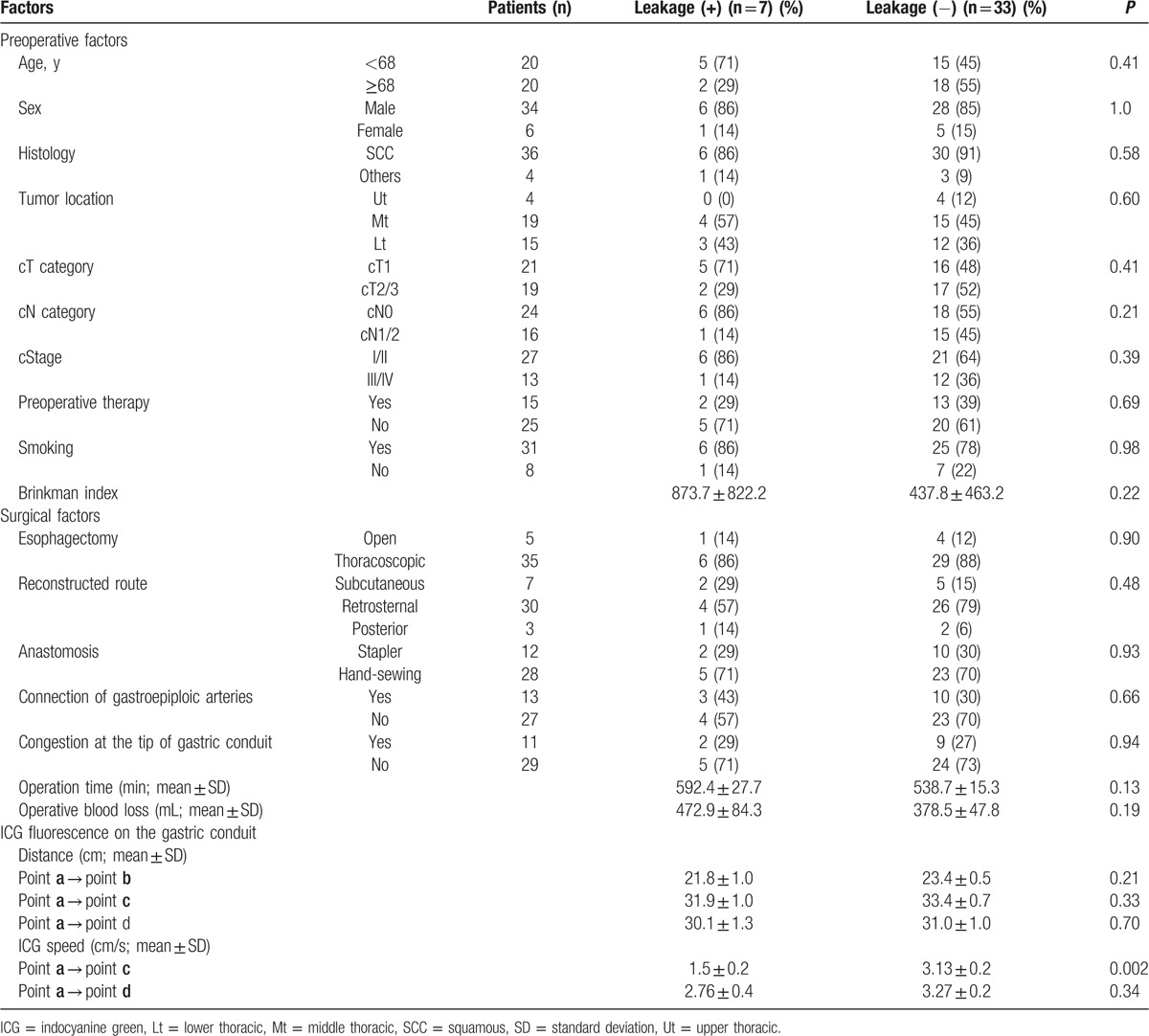
Comparison of clinical factors and ICG fluorescence stream on the gastric conduit wall according to the anastomotic leakage.

Based on the results of the univariate analysis, we selected the flow speed of ICG fluorescence on the gastric conduit wall as a parameter for multivariate analysis to assess the risk factors of anastomotic leakage after esophagectomy. Reconstructed route, operation time, and intraoperative blood loss, which have been considered as risk factors of anastomotic leakage, were also included in the analysis (Table 5). Of these, logistic regression analysis determined that the only independent significant predictor of anastomotic leakage was an ICG fluorescence flow speed in the gastric conduit wall of 1.76 cm/s or less (odds ratio, 36.5; 95% confidence interval, 4.02–905.7; *P* = 0.004).

**Table 5:**
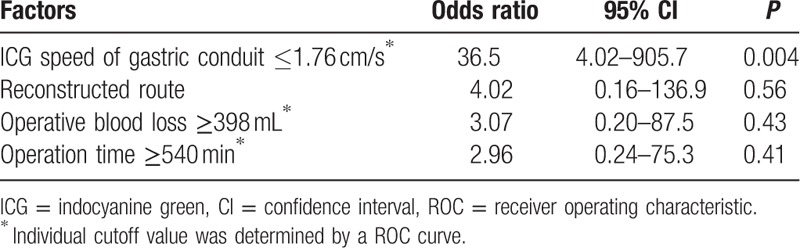
Multivariate analysis for prediction of anastomotic leakage after esophagectomy.

## 4 Discussion

Our study showed that ICG fluorescence can be used to effectively visualize real-time blood flow of the gastric conduit. Previous investigators were uncertain of the significance of visualized ICG fluorescence imaging and hence failed to demonstrate any correlation with esophagogastric anastomotic leakage. In the study, we focused on the blood flow from the pylorus to the terminal end of the ICG fluorescence stream in the gastric conduit wall. We then realized that the ICG fluorescence stream in the gastric conduit wall could be classified into 2 patterns, a simultaneous group where the stream speed was fast and a delayed group where the stream speed was slow. Although tissue blood flow visualized by ICG fluorescence can be impaired by several properties of human blood, medical histories, smoking, and blood pressure during ICG injection,^[24]^ our study demonstrates that these factors have no considerable influence on ICG fluorescence imaging in the gastric conduit wall. Lower concentrations of ICG did not impact the results of ICG fluorescence stream in the study, despite the short half-life of ICG in the blood. These findings indicate that ICG fluorescence imaging could be performed repeatedly in a less-invasive, reproducible assessment of the gastric conduit blood flow.

Contrary to expectations, the flow speed of ICG fluorescence in the gastric conduit wall was not correlated with the connection of right and left gastroepiploic arteries,^[25]^ but with intraoperative blood loss. These findings may be explained by potential reduction in the flow of the capillary vessels in the peripheral tissues during ongoing intraoperative blood loss, since it is important to maintain blood perfusion in the vital organs. In the study, we evaluated the ICG fluorescence imaging before anastomosis. Even though the gastric conduit was placed on the anterior chest wall as assuming the anastomosis, ICG fluorescence might be changed after anastomosis. To dispel this concern, the ICG fluorescence imaging was repeatedly measured before and after anastomosis when the reconstruction route was selected via subcutaneous, and we found that the flow speed of ICG fluorescence after anastomosis was equaled before anastomosis (data not shown).

Anastomotic leakage was not correlated with the reconstructed route and anastomotic technique, but was significantly correlated with the flow speed of ICG fluorescence in the gastric conduit wall. The ROC curve demonstrated that the slower the ICG fluorescence stream flowed, the higher the risk of anastomotic leakage became. Furthermore, no anastomotic leakage occurred in the simultaneous group. Zehetner et al^[26]^ recently demonstrated that anastomotic leakage might frequently occur when esophagogastric anastomosis was placed in the area of no ICG fluorescence. Our anastomosis was placed at the area in which ICG fluorescence was observed in all the patients. These findings strongly recommend that sufficient blood perfusion of the gastric conduit wall is critical to the thorough tissue healing at the anastomosis site. We evaluated the flow speed of ICG fluorescence in the gastric conduit wall, in difference to other investigators who have previously assessed the intensity of ICG fluorescence for quantitative analysis.^[27,28]^ When compared with our assessment, their methods may differentiate between inflow impairment and outflow impairment of the gastric conduit wall. In our study, the flow speed of ICG fluorescence in the gastric conduit wall showed no correlation with connection of right and left gastroepiploic arteries and congestion at the tip of the gastric conduit, but had high sensitivity and specificity to predict anastomotic leakage; therefore, our method could evaluate the comprehensive blood flow including the inflow and outflow of the gastric conduit wall.

The number of patients was limited in our study. To address this, we are now prospectively enrolling patients for further assessment. In addition to the flow speed of the ICG fluorescence stream, there may be other factors that associate with anastomotic leakage, particularly since half of the patients in the delayed group did not suffer from anastomotic leakage. To avoid anastomotic leakage in patients where the ICG fluorescence speed is slow, additional microvascular anastomosis of the gastric conduit might be a useful surgical intervention. It will be important to investigate surgical techniques that can increase the blood flow of the gastric conduit and to locate the exact anastomotic point in which blood perfusion is sufficient for anastomotic healing.

## 5 Conclusion

This preliminary study demonstrates that near-infrared fluorescence using ICG is a promising intraoperative system for assessment of gastric conduit wall blood flow and can predict anastomotic leakage after esophagectomy. The ICG fluorescence flow speed in the gastric conduit wall may be affected by the network of capillary vessels of this tissue. Although there are no system to help surgeons judge the area with appropriate blood perfusion in the gastric conduit, ICG fluorescence has scope to be used for determination of the optimal anastomotic site in the gastric conduit in real-time surgical navigation.
